# Urban-rural disparities in child nutrition-related health outcomes in China: The role of *hukou* policy

**DOI:** 10.1186/s12889-015-2517-4

**Published:** 2015-11-23

**Authors:** Hong Liu, John A. Rizzo, Hai Fang

**Affiliations:** China Economics and Management Academy, Central University of Finance and Economics, Beijing, 100081 China; Department of Economics and Department of Preventive Medicine, Stony Brook University, Stony Brook, 11794 USA; China Center for Health Development Studies, Peking University, 38 Xueyuan Road, Haidian District, PO Box 505, Beijing, 100191 China

**Keywords:** Urban/rural, Nutrition, Child, China

## Abstract

**Background:**

*Hukou* is the household registration system in China that determines eligibility for various welfare benefits, such as health care, education, housing, and employment. The *hukou* system may lead to nutritional and health disparities in China. We aim at examining the role of the *hukou* system in affecting urban-rural disparities in child nutrition, and disentangling the institutional effect of *hukou* from the effect of urban/rural residence on child nutrition-related health outcomes.

**Methods:**

This study uses data from the China Health and Nutrition Survey 1993–2009 with a sample of 9616 children under the age of 18. We compute height-for-age *z*-score and weight-for-age *z*-score for children. We use both descriptive statistics and multiple regression techniques to study the levels and significance of the association between child nutrition-related health outcomes and *hukou* type.

**Results:**

Children with urban *hukou* have 0.25 (*P* < 0.01) higher height *z*-scores and 0.15 (*P* < 0.01) higher weight *z*-scores than children with rural *hukou*, and this difference by urban vs. rural *hukou* status is larger than the difference in height and weight (0.23 and 0.09, respectively) by urban vs. rural residence. Controlling for place of residence, children with urban *hukou* had 0.18 higher height *z*-scores and 0.17 (*P* < 0.01) higher weight *z*-scores than children with rural *hukou*.

**Conclusions:**

The *hukou* system exacerbates urban-rural disparities in child nutrition-related health outcomes independent of the well-known disparity stemming from urban-rural residence. Fortunately, however, child health disparities due to *hukou* have been declining since 2000.

## Background

Health disparities between urban and rural residents pose serious problems for many countries in the world [1–3]. China also has striking urban-rural health differences for both adults [4, 5] and children, the latter of who are more vulnerable to malnutrition and infectious diseases [6]. It is crucial to understand the nature of these urban-rural health disparities and improve population health by better targeting resources. Consistent with the literature on other countries, previous studies in China focus mainly on health disparities due to living locations (urban vs. rural). However, this literature has not considered health disparities associated with China’s specific institution restricting internal migration—the so-called rural–urban dual *hukou* system. The objective of this paper is to examine the role of the *hukou* system in affecting urban-rural disparities in child nutrition, and to disentangle the institutional effect of *hukou* from the effect of residence on child nutrition-related health outcomes.

*Hukou* is the household registration system in China created in 1955 to restrict internal population movement, especially rural-to-urban migration [7]. Two types of *hukou* exist in China: rural *hukou* (also called agricultural *hukou*) and urban *hukou* (also called non-agricultural *hukou*). *Hukou* status was assigned to each child at birth mainly according to mothers’ *hukou* status until 1998. Since 1998, children’s *hukou* status can be inherited from either their mother or father. There are limited opportunities for a rural *hukou* holder to change to an urban *hukou*, such as formal employment by state-owned enterprises, enrollment in colleges or universities, demobilization from military services, and promotion to senior administrative jobs. Even if an individual with rural *hukou* resides in urban area, however, he or she is not permitted to change to urban *hukou* status without legal permission. Importantly, one’s particular *hukou* status affects eligibility for different welfare benefits, such as health care, education, labor, housing, and social subsidies [8, 9].

How might the *hukou* system contribute to urban-rural disparities of child nutrition-related health outcomes in China? First, China has seen accelerating internal migration across the entire country, growing from 50 million in 1990 to 236 million in 2012 (about 18 % of the Chinese population). Due to their rural *hukou* status, rural-to-urban migrants are considered to be temporary employees in urban areas, and as such are not entitled to many welfare benefits and community services available to their urban counterparts. For example, the most serious barrier created by the *hukou* system is that migrant children lack access to public schools in urban areas. Due to these restrictions and poor housing conditions in urban areas, all members of a rural household cannot relocate to cities together [10]. An estimated 23 million children less than 14 years of age are left behind, living without parents, but with grandparents or relatives in rural villages [11]. Studies show that despite the income-enhancing effects of migration, children who are left behind have less health care access, lower intake of essential nutrients, worse physical development, and lower health-related quality of life [12–15].

Another important restriction related to the *hukou* system is that there are currently separate health insurance systems designed for people with urban and rural *hukou* status in China, which differ in financing levels and insurance benefits. Urban health insurance (i.e., employee-based health insurance for the urban employed population or retirees, and urban resident health insurance for non-employed urban *hukou* holders including children) typically offers better health coverage, protection, and reimbursement than rural health insurance. Rural health insurance (referred to in China as the New Cooperative Medical Scheme or NCMS), is a public health insurance program operated at the county level. People with rural *hukou* are expected to participate in the local NCMS and to seek health services from specific providers mostly within their home county. Moreover, most health resources are located in urban China, which are usually considered out-of-network for NCMS enrollees. For example, in 2012, there were 3.9 physicians per 1000 people in urban China, but only 1.4 physicians per 1000 people in rural China. Children with rural *hukou*, no matter whether they are left behind or migrate to urban areas with their parents, are unable to use their NCMS benefits in urban areas [16, 17], but must obtain care in rural areas that offer fewer health care resources. These barriers to access may have potentially important implications for their incidence of diseases and thus health and nutritional status. Childhood malnutrition is a serious health problem which is significantly associated with long-term developmental deficits and high child mortality. Recent statistics reveal that the child (5 years and younger) mortality rate in urban areas was 5.9 % in 2012, but almost three times higher—16.2 %—in rural areas [18]. The most important reason for child mortality is malnutrition.

Motivated by the above facts, the present study seeks to analyze whether urban/rural residence and/or urban/rural *hukou* lead to the rural–urban disparities in nutrition-related health outcomes in China. The previous literature on health disparities in China usually confounds the impacts of urban/rural location and *hukou*, and only focuses on the health effects of urban/rural residence, ignoring the institutional effect of the *hukou* system. Yet quantifying the individual effects of geographic location and the *hukou* system on health disparities has important public policy implications. If these disparities only reflect differences in the availability of adequate nutrition and health resources across urban/rural locations, the policy implication would be to increase public healthcare facilities and access to adequate nutrition in rural areas. But if these disparities are mainly due to the urban/rural *hukou*, the fragmented health insurance structure as well as internal migration control may need to be reformed.

It is quite possible that *both* residential location and *hukou* status play important roles. Moreover, these two factors may work interactively. To examine this issue, we will test for interaction effects between urban/rural location and urban/rural *hukou*. One hypothesis is that living in urban areas may reduce the negative effects of rural *hukou* on child nutrition-related health outcomes. For example, rural-to-urban migrant children who live with their parents may have better nutrition compared to children who are left behind in rural areas, since incomes in the urban areas are much higher. In addition, we employ longitudinal data to examine whether these urban and rural disparities had changed from 1993 to 2009.

## Methods

### Data

We use data from the China Health and Nutrition Survey (CHNS) conducted by the Carolina Population Center, the US National Institute of Nutrition and Food Safety, and the Chinese Center for Disease Control and Prevention. The CHNS is an ongoing longitudinal survey from 1989 to 2009 covering 9 provinces (Guangxi, Guizhou, Heilongjiang, Henan, Hubei, Hunan, Jiangsu, Liaoning, and Shandong) that differ considerably in terms of geography and economic development and include approximately 45 % of China’s total population. In each sample province, counties were stratified into three income levels and then a weighted sampling technique was employed to select four counties randomly. The provincial capital and a low-income city are included when feasible in each province. The primary sampling units of CHNS are urban neighborhoods and suburban villages within the sampled cities and townships and villages within the sampled counties. The overall survey in each wave includes approximately 4400 households and 19,000 individuals. The response rate was quite high for each wave of the survey, on average 88 % at the individual level and 90 % at the household level [19].

#### Ethic approval

China Health and Nutrition Survey data are publicly available and the researchers from the Carolina Population Center had received ethic approval the University of North Carolina at Chapel Hill.

The CHNS survey has collected eight waves of data in 1989, 1991, 1993, 1997, 2000, 2004, 2006, and 2009,^1^ but the *hukou* information has been only asked in waves since 1993. Thus, in this analysis we use CHNS 1993–2009, and focus on the sample of children under the age of 18 in each wave. The study sample is a pooled cross sectional data set with 9616 observations, including 2481 observations in 1993, 2107 observations in 1997, 1774 observations in 2000, 1291 observations in 2004, and 1006 observations in 2006, and 957 in 2009.

### Variables

The CHNS records height and weight for each respondent, measured by a physician, nurse, health worker or other health professional, which avoids recall bias associated with self-reported data. To measure nutrition-related health outcomes, we construct two anthropometric measures: height-for-age *z*-score and weight-for-age *z*-score, using the reference standards of the World Health Organization (WHO) growth chart. The *z*-scores are calculated as the difference between actual height (weight) and mean height (weight) divided by the standard deviation in the reference children population of same age and gender, which have been widely used as important outcome measures of child nutritional status in the literature [20–23]. According to WHO (2006) and the previous studies [24, 25], height-for-age *z*-score is a good indicator measuring the long-run health and nutritional experience of all children under 18 years of age, while weight-for-age *z*-score reflects short-term changes in child nutritional status but is only a suitable measure for children under 10 years of age. Therefore, we compute height-for-age *z*-scores for all children from 0 to 17.99 and weight-for-age *z*-score for children aged 0 to 10.

Based on a survey question on respondents’ current household registration status, we construct one main explanatory variable indicating whether a child has an urban or a rural *hukou* (urban *hukou* = 1, rural *hukou* = 0). A second key explanatory variable is a dichotomous variable indicating whether a child resides in an urban or a rural area. In contrast to the urban definition in the US that is a densely populated area consisting of 50,000 or more people [26], urban location as defined by the National Bureau of Statistics of China is an urban district, city or town with a population density greater than 1500/km^2^. Following this administrative definition, we classify city neighborhoods and county town neighborhoods as urban areas and classify suburban and rural villages as rural areas in CHNS.

The other covariates relevant to child nutrition-related health outcomes include child and household characteristics. Child demographic characteristics include age, gender, Han nationality (Han is the largest ethnic group in China), student status, whether the child has health insurance coverage, and whether the child lives with his/her mother, father or grandparents at the survey time. Household measures include household income per capita, gender of household head, household size, and parents’ demographic and socioeconomic characteristics. The specific parents’ variables used in the analysis are parents’ age, height, BMI, education, smoking status and alcohol consumption. Both smoking status and alcohol consumption are measured as binary variables indicating whether the mother or father smokes cigarettes at the survey time, and whether the mother or father has drunk any alcoholic beverage in the previous year.

In addition, we include provincial binary variables to control for regional differences that may be associated with child health and nutritional status. Five wave binary indicators for survey periods are included to capture time trend effects as well.

### Statistical analyses

In the analysis, we use both descriptive statistics and multiple regression techniques to study the levels and significance of the association between child nutrition-related health outcomes and *hukou* type. We use the chi-square test for dichotomous variables and Student’s *t*-test for continuous variables. The descriptive analysis compares outcomes and other confounding variables by residence and *hukou* status. Then we employ a multivariate regression approach to examine the impact of *hukou* type on child nutrition-related health outcomes, after controlling for place of residence and other child and parents’ sociodemographic characteristics, using pooled CHNS data for 1993–2009. Ordinary least squares estimation is conducted for the continuous nutritional outcome variables measured by height-for-age and weight-for-age *z*-scores. There are four model specifications for each outcome variable. Models 1 and 2 include a binary variable for urban residence and another one for urban *hukou*, respectively, to separately investigate differential effects of urban residence and urban *hukou* on child health and nutritional outcomes. Model 3 controls for both *hukou* status and place of residence. Model 4 extends Model 3 to include an interaction term between *hukou* status and place of residence. Moreover, we also perform the multivariate regression analyses for different time periods and regions and examine whether health disparities associated with residence and/or *hukou* changed from 1993 to 2009, or varied between coastal and interior regions.

## Results

### Bivariate results

Table 1 displays descriptive statistics for the full study sample and separately for children with different residence-*hukou* status. Approximately 25 % of the sample resides in urban areas. But 12.4 % of these urban residents have rural *hukou*, and 13.7 % of those living in rural areas have urban *hukou*. Thus, place of residence does not define one’s *hukou* status in many cases. Approximately one-third of children have urban *hukou* in our sample. Our previous study finds significant differences in health and nutritional status between children with urban and rural residence [6]. However, even for those children with the *same* residence, most of the variables listed in Table 1 differ significantly by *hukou* status. For example, children with urban *hukou* had significantly higher height-for-age *z*-scores than those with rural *hukou*, no matter whether they lived in urban or rural areas. The difference in weight-for-age *z*-score by *hukou* status is also highly significant (*p* < 0.001) for children with rural residence, but only significant at the 10 % level for those with urban residence.

**Table 1:** Name and descriptive statistics for study variables by residency and *hukou* status

		Urban Residents			Rural Residents		
Variable	Full	Urban *hukou*	Rural *hukou*		Urban *hukou*	Rural *hukou*	
Sample Size	*N* = 9616	*N* = 2103	*N* = 298		*N* = 985	*N* = 6230	
*Health and nutritional status*							
Height-for-age *z*-score	−0.83	−0.36	−0.77	***	−0.51	−1.04	***
Weight-for-age *z-*score^a^	−0.56	−0.18	−0.39	*	−0.19	−0.73	***
*Control variables*							
Girl	0.47	0.47	0.41	*	0.49	0.47	
Han	0.85	0.89	0.94	***	0.91	0.82	***
Student	0.79	0.84	0.75	***	0.85	0.76	***
Health insurance	0.27	0.29	0.23	**	0.31	0.26	***
Child age							***
0–5	0.08	0.07	0.08		0.05	0.09	
6–11	0.46	0.43	0.41		0.45	0.47	
12–17	0.47	0.50	0.51		0.50	0.45	
Living with mother	0.90	0.86	0.90		0.90	0.91	
Living with father	0.93	0.91	0.93		0.93	0.93	
Living with grandparents	0.27	0.31	0.18	***	0.32	0.26	***
Household head is male	0.84	0.78	0.81		0.80	0.87	***
Household size	4.38	3.84	4.42	***	4.14	4.60	***
Household income (in 2009 value)				**			***
< 10,000	0.28	0.26	0.34		0.17	0.31	
10,001–20,000	0.34	0.34	0.28		0.31	0.34	
20,001–30,000	0.17	0.17	0.17		0.21	0.16	
30,000 and above	0.21	0.23	0.20		0.31	0.19	
Mother’s age	37.81	38.24	38.39		37.59	37.67	
Mother’s height	155.86	157.52	158.01		156.97	155.04	***
Mother’s BMI	22.55	22.61	23.10	**	22.85	22.46	***
Mother’s education				***			***
< primary school	0.23	0.07	0.16		0.12	0.31	
Primary school	0.23	0.11	0.26		0.14	0.29	
Lower middle school	0.34	0.35	0.44		0.44	0.32	
Upper middle school	0.17	0.40	0.14		0.25	0.08	
College	0.02	0.08	0.00		0.04	0.00	
Mother smokes	0.02	0.01	0.00		0.01	0.03	***
Mother drinks	0.10	0.12	0.06	***	0.09	0.10	
Father’s age	39.34	40.34	39.14	***	39.29	39.04	
Father’s height	166.38	168.32	167.58	*	167.90	165.47	***
Father’s BMI	22.53	23.38	23.93	***	23.27	22.08	***
Father’s education				***			***
< primary school	0.09	0.03	0.10		0.05	0.12	
Primary school	0.21	0.09	0.18		0.14	0.27	
Lower middle school	0.43	0.36	0.38		0.45	0.45	
Upper middle school	0.23	0.40	0.32		0.30	0.16	
College	0.03	0.13	0.02		0.06	0.00	
Father smokes	0.67	0.64	0.65		0.67	0.67	
Father drinks	0.69	0.72	0.74		0.68	0.68	
Presence of mother	0.91	0.89	0.91		0.91	0.92	
Presence of father	0.88	0.84	0.87		0.88	0.90	*
Province				***			***
Liaoning	0.08	0.07	0.03		0.18	0.07	
Heilongjiang	0.09	0.11	0.00		0.03	0.10	
Jiangsu	0.10	0.12	0.01		0.14	0.09	
Shandong	0.09	0.09	0.22		0.13	0.07	
Henan	0.11	0.11	0.19		0.03	0.12	
Hubei	0.12	0.09	0.14		0.11	0.12	
Hunan	0.11	0.11	0.27		0.27	0.08	
Guangxi	0.15	0.13	0.06		0.07	0.17	
Guizhuo	0.16	0.16	0.08		0.04	0.18	
Wave				**			***
1993	0.26	0.23	0.31		0.21	0.27	
1997	0.22	0.21	0.21		0.19	0.23	
2000	0.18	0.20	0.17		0.20	0.18	
2004	0.13	0.14	0.08		0.16	0.13	
2006	0.10	0.11	0.11		0.12	0.10	
2009	0.10	0.10	0.11		0.12	0.10	

*χ* ^2^ test for category variables and *t*-test for continuous variables. * *p* < 0.10, ** *p* < 0.05, *** *p* < 0.01

*Hukou* status is also associated with significant differences in socioeconomic and demographic characteristics. Compared with urban *hukou* children, rural *hukou* children are less likely to be students, have lower rates of health insurance coverage and larger household size, but are less likely to live with grandparents. Parents of children with urban *hukou* have higher incomes and better educational attainment than their rural counterparts. Most of these differences are statistically significant with *P* < 0.001. In rural areas, parents of urban *hukou* children were significantly taller than their rural counterparts, but this height difference between parents is not significant in urban areas.

### Multivariate results

Table 2 presents the results of the multivariate analysis for child height-for-age *z*-scores using the total sample of children under 18 years of age, and Table 3 reports the results for weight-for-age *z*-scores using the sample of children aged 0–10. In these estimates, we pool the six waves of CHNS data and control for the possible confounding effect of the covariates, including binary indicators for each wave and each province.

**Table 2:** Multivariate analyses of children’s height-for-age *z*-score using pooled CHNS data 1993–2009

	Height-for-age *z*-score (Children aged 0–17.99)			
Variables	(1)	(2)	(3)	(4)
Urban residency	0.23***		0.12***	0.01
Urban hukou		0.25***	0.18***	0.14***
Urban residency × urban hukou				0.16**
Health insurance	0.15***	0.15***	0.15***	0.15***
Child age: 0–5	reference	reference	reference	reference
Child age: 6–11	−0.04	−0.06	−0.06	−0.06
Child age: 12–17	−0.19***	−0.21***	−0.20***	−0.20***
Girl	−0.02	−0.02	−0.02	−0.02
Han	0.00	−0.00	−0.01	−0.01
Student	0.06*	0.05*	0.05*	0.05*
Living with mother	−0.09	−0.12	−0.12	−0.12
Living with father	0.32***	0.30***	0.30***	0.30***
Living with grandparents	0.13***	0.12***	0.11***	0.11***
Household income: <10,000	reference	reference	reference	reference
Household income: 10,001–20,000	0.07***	0.06**	0.07**	0.07**
Household income: 20,001–30,000	0.15***	0.14***	0.14***	0.14***
Household income: >30,000	0.14***	0.12***	0.13***	0.13***
Household head is male	−0.11**	−0.09*	−0.09*	−0.09*
Household size	−0.05***	−0.05***	−0.05***	−0.05***
Mother’s age	0.00	0.00	0.00	0.00
Mother’s height	0.04***	0.04***	0.04***	0.04***
Mother’s BMI	0.04***	0.04***	0.04***	0.04***
Mother’s education				
< primary school	reference	reference	reference	reference
Primary school	−0.03	−0.03	−0.04	−0.04
Middle school	0.03	0.01	0.00	0.01
High school	0.09**	0.06	0.05	0.05
College	0.05	0.01	−0.01	−0.01
Father’s age	0.01***	0.01**	0.01**	0.01**
Father’s height	0.04***	0.04***	0.04***	0.04***
Father’s BMI	0.02***	0.02***	0.02***	0.02***
Father’s education				
< primary school	reference	reference	reference	reference
Primary school	0.08*	0.07*	0.07*	0.07
Middle school	0.09**	0.08*	0.08*	0.07*
High school	0.11**	0.10**	0.10**	0.09**
College	0.33***	0.31***	0.30***	0.29***
N	9745	9616	9616	9616

(i) * *p* < 0.10, ** *p* < 0.05, *** *p* < 0.01; (ii) other covariates include indicators for whether mother or father is missing in the survey, provincial dummies, wave dummies and a constant, which are not reported here

**Table 3:** Multivariate analyses of children’s weight-for-age *z*-score using pooled CHNS data 1993–2009

	Weight-for-age *z*-score (Children aged 0–10)			
Variables	(1)	(2)	(3)	(4)
Urban residency	0.09**		−0.00	−0.08
Urban *hukou*		0.15***	0.16***	0.13**
Urban residency × urban *hukou*				0.11
Health insurance	0.09**	0.09**	0.09**	0.09**
Child age: 0–5	reference	reference	reference	reference
Child age: 6–11	−0.35***	−0.36***	−0.36***	−0.36***
Child age: 12–17	0.00	0.00	0.00	0.00
Girl	−0.15***	−0.14***	−0.14***	−0.14***
Han	−0.02	−0.03	−0.03	−0.03
Student	−0.03	−0.04	−0.04	−0.04
Living with mother	0.06	0.10	0.10	0.11
Living with father	0.23	0.16	0.16	0.16
Living with grandparents	0.11**	0.09**	0.09**	0.09**
Household income: <10,000	reference	reference	reference	reference
Household income: 10,001–20,000	−0.00	0.00	0.00	0.00
Household income: 20,001–30,000	−0.00	−0.00	−0.00	−0.00
Household income: >30,000	0.01	0.01	0.01	0.01
Household head is male	0.05	0.05	0.05	0.05
Household size	−0.03*	−0.03	−0.03	−0.03
Mother’s age	−0.00	0.00	0.00	0.00
Mother’s height	0.03***	0.03***	0.03***	0.03***
Mother’s BMI	0.07***	0.07***	0.07***	0.07***
Mother’s education				
< primary school	reference	reference	reference	reference
Primary school	−0.11*	−0.11*	−0.11*	−0.12*
Middle school	0.02	−0.00	−0.00	−0.00
High school	0.05	0.02	0.02	0.02
College	0.36**	0.30*	0.31*	0.30*
Father’s age	0.00	0.00	0.00	0.00
Father’s height	0.03***	0.03***	0.03***	0.03***
Father’s BMI	0.06***	0.06***	0.06***	0.06***
Father’s education				
< primary school	reference	reference	reference	reference
Primary school	−0.11	−0.12	−0.12	−0.12
Middle school	−0.09	−0.10	−0.10	−0.10
High school	0.04	0.03	0.03	0.03
College	0.21	0.18	0.18	0.18
N	3575	3525	3525	3525

(i) * *p* < 0.10, ** *p* < 0.05, *** *p* < 0.01; (ii) other covariates include indicators for whether mother or father is missing in the survey, provincial dummies, wave dummies and a constant, which are not reported here

In Table 2, the results in columns (1) and (2) reveal significant differences in height-for-age *z*-scores between children living in urban and rural areas as well as between children with urban and rural *hukou*. Children with urban *hukou* have 0.25 higher height-for-age *z*-scores (i.e., 1.59 centimeters for a 10-year-old boy,^2^ for example) than children with rural *hukou*, while the difference by rural–urban residence is 0.23 *z*-scores (i.e., 1.47 centimeters for a 10-year-old boy).

Column (3) controls for both *hukou* type and place of residence to separate the institutional effect of *hukou* type from the spatial effect of residence on child nutrition-related health outcome. These results indicate that, independent of the advantage of urban residence, urban *hukou* status has an additional positive and significant effect on child height-for-age *z*-score. Controlling for place of residence, children with urban *hukou* had 0.18 higher height-for-age *z*-scores (i.e., 1.15 centimeters in height for a 10-year-old boy) than children with rural *hukou*. In contrast, the net advantage in height-for-age *z*-scores for those children living in urban areas is only 0.12 (i.e., 0.76 centimeters in height for a 10-year-old boy), net of *hukou* status.

In column (4) of Table 2, we add an interaction term between *hukou* status and residence. The results reveal that, among the sample of children under 18 years of age, the most disadvantaged children were those with rural *hukou*, regardless of whether they lived in urban or rural areas, That is, children with rural *hukou* do just as poorly whether they live in rural or urban areas. In contrast, the most advantaged children were those who had urban *hukou* and lived in urban areas. The difference between these two groups is approximately 0.30 height-for-age *z*-scores (i.e., 1.91 centimeters in height for a 10-year-old boy). Among children with urban *hukou*, those living in rural areas had 0.16 significantly lower height-for-age *z*-scores (i.e., 1.02 centimeters in height for a 10-year-old boy) than those living in urban areas.

In the analysis for weight-for-age *z*-scores using the sample aged 0–10 in Table 3, we find similar results to those reported in Table 2. Specifically, when we control for residence and *hukou* type in columns (1) and (2) of Table 3, respectively, we find that the weight difference between children with urban and rural *hukou* is 0.15 *z*-scores, which is larger than the difference (0.09 *z*-scores) between children with urban and rural residence. Moreover, as shown in column (3), the positive effect of urban *hukou* on weight-for-age *z*-scores is robust to the inclusion of residence. The difference between children with urban and rural *hukou* is still 0.16 weight-for-age *z*-scores (i.e., approximately 0.81 kilograms for a 10-year-old boy, for example) and significant at the 1 % level, whereas the difference by residence disappears after controlling for *hukou* status. In column (4), the interaction term between *hukou* status and residence is insignificant, and we again find that only *hukou* status, not residence, leads to significant differences in weight-for-age z-scores.

### Inter-temporal and regional effects

To study the heterogeneous effects of *hukou* status on child health and nutritional status in China, we also estimate multivariate regression models for different time periods and regions. The coefficients on key explanatory variables are reported in Table 4.

**Table 4:** Multivariate analyses by periods and by regions

	Height-for-age *z*-score		Weight-for-age *z*-score	
	(1)	(2)	(3)	(4)
	1993–1997	2000–2009	1993–1997	2000–2009
Only control for residence				
Urban residency	0.25***	0.19***	0.11*	0.04
Only control for Hukou				
Urban hukou	0.33***	0.15***	0.21***	0.05
Without interaction				
Urban residency	0.07	0.15***	−0.04	0.03
Urban hukou	0.29***	0.08*	0.24***	0.04
With interaction				
Urban residency	−0.12	0.15*	−0.11	0.04
Urban hukou	0.20***	0.06	0.20**	0.04
Urban residency × urban hukou	0.29***	0.00	0.11	−0.02
N	4588	5028	1998	1527
	Coastal Regions	Non-Coastal Regions	Coastal Regions	Non-Coastal Regions
Only control for residence				
Urban residency	0.18***	0.26***	0.08	0.10*
Only control for Hukou				
Urban hukou	0.32***	0.21***	0.26***	0.10*
Without interaction				
Urban residency	0.00	0.19***	−0.10	0.08
Urban hukou	0.32***	0.10**	0.31***	0.05
With interaction				
Urban residency	−0.21*	0.07	−0.39*	0.02
Urban hukou	0.27***	0.04	0.26***	0.02
Urban residency × urban hukou	0.27*	0.19**	0.36	0.10
N	3434	6182	1180	2345

(i) * *p* < 0.10, ** *p* < 0.05, *** *p* < 0.01; (ii) other control variables include child’s characteristics as age, gender, nationality, student status, and indicators of living with mother, father, or grandparents; household characteristics as household income, gender of household head, and household size; mother’s characteristics if present in the survey, such as mother’s age, height, BMI, education, and an indicator for the presence of mother in the survey; father’s characteristics if present in the survey, such as father’s age, height, BMI, education, and an indicator for the presence of father in the survey; indicators of provinces; and a constant, which are not reported here

The upper Panel of Table 4 presents the multivariate results for two time periods, 1993–1997 and 2000–2009, separately. The results show that children with urban *hukou* were consistently better than children with rural *hukou* in terms of height-for-age *z*-scores, but these differences decreased from the period 1993–1997 to 2000–2009. Controlling for both *hukou* and residence, we find only a significant positive effect (0.29 *z*-scores, *P* < 0.01) of urban *hukou* but an insignificant effect of urban residence on children’s height-for-age *z*-score in the period 1993–1997; however, in the period 2000–2009 the height disparities due to *hukou* status declined to 0.08 *z*-scores and were only significant at the 10 % level. Moreover, of the children aged from 0 to 10, those with urban *hukou* had 0.21–0.24 greater weight-for-age *z*-scores than those with rural *hukou* in the period 1993–1997, while this weight disparity disappeared in the period 2000–2009.

In the lower Panel of Table 4, we compare the results for the relatively more developed coastal regions and the less developed non-coastal regions. These results indicate that the differences in both height and weight *z*-scores by *hukou* status are more pronounced for children in coastal regions than those in non-coastal regions. Controlling for both residence and *hukou* status, there are only significant height and weight disparities by *hukou* status but no significant disparities by place of residence in coastal regions. However, in non-coastal regions there are both significant height disparities by *hukou* and by residence.

## Discussion

The present study finds that overall, *hukou* status has a stronger association with disparities in child height and weight *z*-scores than rural/urban residence in China. The previous literature that takes little account of the effects of *hukou* policy in China may fail to capture the whole picture of urban-rural differences in child nutritional status and associated health outcomes. Controlling for both *hukou* and residence, we find that the net advantages of urban *hukou* on child height and weight are 0.18 and 0.16 *z*-scores (i.e., 1.15 centimeters in height and 0.81 kilograms for a 10-year-old boy, for example). The magnitude of these effects is striking, as the literature show that the disparities of child nutritional status by urban/rural residence are about 0.28–0.45 height-for-age *z*-score and 0.19 weight-for-age *z*-score in China [6, 22]. Moreover, previous studies focusing on the determinants of child nutritional status in China find that only-child status is associated with 0.12 units higher height-for-age *z*-score [23]; paternal job loss reduces height-for-age and weight-for-age *z*-scores by 0.33–0.34 units [27]; and a water quality improvement program leads to an increase in child height by 0.962 centimeters in rural China [28]. Thus, this study adds to the body of literature by showing that rural *hukou* has an additional-and indeed a stronger-negative impact on child nutrition-related health outcomes, independent of the well-established nutritional disadvantage of rural residence.

As coastal China has high levels of urbanization and fewer disparities in income and health resources by urban-rural residence, we find that the urban-rural health disparities are mainly associated with *hukou* in coastal China, but associated with both residence and *hukou* in inland China. These findings suggest that allocating more health resources to rural areas may not solve urban-rural health disparities completely, as the institutional hurdles associated with *hukou* persist in China.

This study has some limitations that must be acknowledged. First, due to data limitations, we use anthropometric indicators to measure child nutrition-related health outcome. But child health status is multidimensional and nutritional status is only one dimension, albeit an important one. However, none of the existing studies has ever examined the effect of *hukou* policy on health outcomes, including child nutritional outcome. Our findings are consistent with the broader literature on the *hukou* system in China, which suggests that the *hukou* system is the major cause of rural–urban disparities in social and economic outcomes at the individual level, such as income, education, healthcare benefits and employment [7, 8]. Future work is needed to explore more health disparities associated with the hukou policy.

Second, we are unable to identify the place of origin and migration status of parents and children in the CHNS data, and cannot control for this effect in the multivariate analysis. The *hukou* system not only splits the population into urban and rural *hukou* holders, but also links people with their place of origin. Migrants without local *hukou*, especially in big cities, are not entitled to the full spectrum of welfare programs provided by local governments [9]. With an increasing trend of internal migration in China since the 1990s, it is important to further evaluate the distinction between local and non-local *hukou* as a critical factor in predicting children’s health status in future research, when the data become available.

The main reason for child health disparities due to *hukou* is that *hukou* restricts access to health care, education, employment and other welfare benefits for rural people. There are two channels linking *hukou* and children’s nutritional status. The first channel is that rural children are usually left behind due to the *hukou* restrictions on their eligibility for local compulsory education, as well as high urban living costs given their migrant parents’ limited income. Rural parents, who move to long-distance destination cities or have lengthy commutes to and from work, may spend less time on food preparation and child healthcare [13]. Left-behind children are living with their grandparents or attend boarding schools in rural China. They are more likely to suffer from malnutrition problems because of limited health knowledge of grandparents, poor food quality in boarding schools [29], or lack of access to safe drinking water in rural villages [24, 28].

The second channel is through the *hukou*-related health insurance types. Rural *hukou* people are only eligible for the rural health insurance program, which has much more limited health benefits and lower health reimbursement compared to the urban health insurance program. Moreover, despite an increasing number of rural migrants moving to urban areas for employment in the past two decades, they cannot use their local NCMS in places other than their hometown, and have little access to health insurance programs in the cities where they work. As a result, for example, pregnant migrant women lack access to prenatal care and support, which may have a negative impact on birth outcomes, and in turn, correlate with worse nutritional status of children later in life [30, 31]. Moreover, there are direct barriers to health care access for many rural migrant children in urban China, including lack of regular well-baby checkups and immunization.

Rural children’s lack of access to preventive and curative health services may lead to worse nutrition-related health outcomes due to high incidence and severity of illness [23, 32, 33]. Childhood disease is also associated with reduced dietary intake, poor nutrient absorption, and high needs for nutrients to fight disease, which may lead to depletion of nutritional stores and consequently growth retardation, especially for young children [34–36].

## Conclusions

An encouraging finding is that the child health disparities due to *hukou* have been declining since 2000. This probably reflects the improvement of rural children’s nutrition intake associated with income growth, as well as the separation of some welfare benefits (i.e. primary education) from *hukou* status during the ongoing *hukou* reform. However, our results after the year 2000 still show that child health disparities in term of height are more serious than that in terms of weight. Further efforts are needed to address these child health disparity issues. Fortunately, the Chinese government has recently announced plans to abolish the *hukou* system altogether. Our results attest to the wisdom of this new policy. An additional policy initiative to reduce if not eliminate rural/urban health disparities is to improve the rural health insurance system substantially and develop a more comprehensive health system offering the same health insurance coverage for all of China’s population.

## Consent

People studied in the China Health and Nutrition survey had given their consent for the data to be published and the analyses using these data to be published. The written consents can be obtained from Carolina Population Center at the University of North Carolina Chapel Hill.

### Availability of data and materials

The data used in this study are China Health and Nutrition Survey and are publicly available from http://www.cpc.unc.edu/projects/china.

## Footnotes

1 The ninth wave of the CHNS data was collected in 2011, but data on child height and weight have not been released yet when the present study was conducted. So we are not able to include 2011 wave of CHNS data.

2 The average height and weight for a 10-year-old boy are 137.8 centimeters and 31.2 kilograms according to the WHO growth chart.

## References

[1] Agency for Healthcare Research and Quality. Fact sheet: Health care disparities in rural areas. AHRQ 2005. Publication No. 05-P022. Available: http://archive.ahrq.gov/research/ruraldisp/ruraldispar.htm.

[2] Pong RW, DesMeules M, Lagace C (2009). Rural-urban disparities in health: How does Canada fare and how does Canada compare with Australia?. Aust J Rural Health.

[3] Van de Poel E, O’Donnell O, Van Doorslaer E (2007). Are urban children really healthier**?** Evidence from 47 developing countries. Soc Sci Med.

[4] Liu Y, Hsiao WC, Li Q, Liu X, Ren M (1995). Transformation of China’s rural health care financing. Soc Sci Med.

[5] Fang H, Chen J, Rizzo JA (2009). Explaining urban-rural health disparities in China. Med Care.

[6] Liu H, Fang H, Zhao Z (2013). Urban-rural disparities of child health and nutritional status in China from 1989 to 2006. Econ Hum Biol.

[7] Wu X, Treiman DJ (2004). The household registration system and social stratification in China: 1955–1996. Demography.

[8] Liu Z (2005). Institution and inequality: The hukou system in China. J Comp Econ.

[9] Song Y (2014). What should economists know about the current Chinese hukou system?. China Econ Rev.

[10] de Brauw A, Mu R. Unattended but not undernourished – young children left behind in rural china. IFPRI 2012 Discussion Paper 01191. Available: http://www.ifpri.org/publication/unattended-not-undernourished.

[11] Guo L. Living arrangements of migrants’ left-behind children in China. Paper presented at the Population Association of America 2009 Annual Conference 2009.

[12] Luo J, Peng X, Zong R, Yao K, Hu R, Du Q (2008). The status of care and nutrition of 774 left-behind children in rural areas in China. Public Health Rep.

[13] de Brauw A, Mu R (2011). Migration and the overweight and underweight status of children in rural China. Food Policy.

[14] Wen M, Lin D (2012). Child development in rural China: Children left behind by their migrant parents and children of nonmigrant families. Child Dev.

[15] Jia Z, Shi L, Cao Y, Delancey J, Tian W (2010). Health-related quality of life of “left-behind children”: A cross-sectional survey in rural China. Qual Life Res.

[16] Qiu P, Yang Y, Zhang J, Ma X (2011). Rural-to-urban migration and its implication for new cooperative medical scheme coverage and utilization in China. BMC Public Health.

[17] Lam KK, Johnston JM (2012). Health insurance and healthcare utilisation for Shenzhen residents: A tale of registrants and migrants?. BMC Public Health.

[18] China Health and Family Planning Commission (2013). China Health and Family Planning Statistical Yearbook.

[19] Popkin BM, Du S, Zhai F, Zhang B (2010). Cohort profile: The China Health and Nutrition Survey—monitoring and understanding socio-economic and health change in China, 1989–2011. Int J Epidemiol.

[20] Shen T, Habicht JP, Chang Y (1996). Effect of economic reforms on child growth in urban and rural areas of China. N Engl J Med.

[21] Smith LC, Ruel MT, Ndiaye A (2005). Why is child malnutrition lower in urban than in rural areas? Evidence from 36 developing countries. World Dev.

[22] Chen Z, Eastwood DB, Yen ST (2007). A decade’s story of childhood malnutrition inequality in China: Where you live does matter. China Econ Rev.

[23] Bredenkamp C (2009). Policy-related determinants of child nutritional status in China: The effect of only-child status and access to healthcare. Soc Sci Med.

[24] Mangyo E (2008). The effect of water accessibility on child health in China. J Health Econ.

[25] Chen Y, Li H (2009). Mother’s education and child health: Is there a nurturing effect?. J Health Econ.

[26] US Census Bureau. Statistical Abstract of the United States. US Census Bureau, 2004–2005. Available: http://www.census.gov/prod/2004pubs/04statab/pop.pdf.

[27] Liu H, Zhao Z (2014). Parental job loss and children’s health: Ten years after the massive layoff of the SOEs’ workers in China. China Econ Rev.

[28] Zhang J (2012). The impact of water quality on health: Evidence from the drinking water infrastructure program in rural China. J Health Econ.

[29] Luo R, Shi Y, Zhang L, Liu C, Rozelle S, Sharbono B (2009). Malnutrition in China’s rural boarding schools: The case of primary schools in Shaanxi Province. Asia Pac J Educ.

[30] Binkin NJ, Yip R, Fleshood L, Trowbridge FL (1988). Birth weight and childhood growth. Pediatrics.

[31] Peralta-Carcelen M, Jackson DS, Goran MI, Royal SA, Mayo MS, Nelson KG (2000). Growth of adolescents who were born at extremely low birth weight without major disability. J Pediatr.

[32] Alderman H, Garcia M (1994). Food security and health security: Explaining the levels of nutrition in Pakistan. Econ Dev Cult Change.

[33] Behrman JR, Skoufias E (2004). Correlates and determinants of child anthropometrics in Latin America: Background and overview of the symposium. Econ Hum Biol.

[34] Scrimshaw NS, SanGiovanni JP (1997). Synergism of nutrition, infection, and immunity. Am J Clin Nutr.

[35] Weisz A, Meuli G, Thakwalakwa C, Trehan I, Maleta K, Manary M (2011). The duration of diarrhea and fever is associated with growth faltering in rural Malawian children aged 6–18 months. Nutr J.

[36] Rodríguez L, Cervantes E, Ortiz R (2011). Malnutrition and gastrointestinal and respiratory infections in children: A public health problem. Int J Environ Res Public Health.

